# Indents

**Published:** 1904-03-15

**Authors:** 


### INDENTS.

☞ **Brief Articles of Technical or Scientific Value will receive notice and comment. Contributions invited.**

**Asymmetry.** The two sides of the face are not in accord one with the other. The left side is nearly always the fuller and better formed.

**Erosion.** In cases of erosion make the fillings *concave* on the surface to protect margins from acid secretions, which will then accumulate in the saucer-like depression and expend their effect on the filling material.—F. W. Stephan, in *Review*.

**Obtunding Dentin.** Dry the tooth and apply a saturated solution of tannin in glycerine, rubbing it well over the sensitive surface. It is also serviceable in cavities as well, and there is rarely a return of the sensitiveness.—S. F. Howland, in *Review*.

**To Clean Plaster Bowls.** When plaster has been left in the bowl 'till it becomes hard, pour boiling water in the bowl and allow it to remain for about two minutes. The plaster can then be easily detached, in most cases without the aid of a spatula.—Abraham M. Waas, in *Review*.

**Swage for Adapting Caps to End of Root.** Cut a disk from a rubber band (such as is used for holding books), about the size of the end of the root. Burr a hole through the disk to fit over the post, and with a bur in the engine bore a hole in the end of a piece of wood which is about the size of the end of the post. Place this over the post and use as a swage.—J. Mills, in *Review*.

**Cleaning Impression Trays.** Mercury and whiting rubbed together and applied to impression trays after removing wax, plaster, etc., keeps them looking like new. This suggestion I received from Dr. McCulloch, of Perth, Ont.—A. W. Thornton, in *Review.*

**Overcome Gagging.** Dipping the plate into strong vinegar before inserting it in the patient's mouth will prevent any ordinary case of gagging. In extreme cases, bathe the roof of the mouth with a menthol solution applied on a pledget of cotton.—C. L. Davis, in *Summary.*

**Repairing a Lower Plate.** Drill two small holes through the plate on each side of the fracture, insert binding wire through these and twist the ends together. This will bring the broken parts into correct relationship and hold them while model is being obtained.—A. W. Thornton, in *Review.*

**Cherry Tooth Powder.**

| | |
|---|---|
| Honey, purified | parts 225 |
| Chalk, precipitated | parts 225 |
| Orris, root, powdered | parts 225 |
| Rose leaves, powdered | parts 28 |
| Simple syrup | sufficient |
| Clove oil | drops 30 |
| Mace oil | drops 30 |
| Geranium oil | drops 30 |

**Physiological Tooth-powder.**

℞ Prepared chalk
Carbonate of magnesia ---------- aa ℥ j.
Pulverized orris root.
Precipitated sulphur ---------- aa ℥ ½

Mix, flavor and color to suit taste.

Use a small tooth-brush of suitable shape and style to reach well around the upper third molars and other irregular conditions of the arches.—T. W. Finlayson, in *Dental Record.*

**Silence Aseptic.** The old adage that silence is golden has been changed by a recent investigator into "silence is aseptic." The new form is specially applied to the operating surgeon who is advised to keep his mouth shut during operations so as to avoid risk of infecting the patient. By speaking three hundred words in front of an agar-agar slide and "developing" the latter, an investigator has found over a quarter of a million germs growing thereon. "Speech is septic; silence, aseptic."—*Moral for the talking Dentist.*

**Applying the Rubber Dam over deep Cervical Cavities.** Clean the cavity thoroughly, mix some quick-setting cement and fill, covering all the margins. Anesthetize the gum by use of the hypodermic syringe, and by the time this is accomplished the cement is hard. The rubber dam can now be applied, and the ease with which the clamp can be adjusted will compensate the operator for the time required in placing the cement. This method really saves time for the operator, and renders the operation comparatively painless for the patient. After the rubber dam is adjusted, the cement can be easily removed.—N. L. Burke, Benton Harbor, Mich., in *Dental Review.*

**Plastic Fillings.** Whether of amalgam or oxyphosphates, must have surrounding walls to resist the pressure necessary in packing. If one or more of the walls are missing, they should be supplied by some form of matrix suitable to the case in hand. An "all round" matrix is the best for badly broken down teeth, and when amalgam is used, let it remain until the following day, when it can be removed and the filling shaped and polished. In our hands the "all round" matrix made of German silver, 34 to 36 guage, is most satisfactory. A number of rings can be kept on hand of size for molars and bicuspids so as to avoid delay in soldering for each case which presents. The rigidity of German silver is sufficient, yet it can be readily bent to the proper contour and rigidly kept in position by wedges or red base-plate gutta-percha. An entire amalgam crown can thus be made when the expense of gold must be considered.—*Western Journal.*

**Camphor.** The Japanese government, father of the camphor monopoly, has been greatly worried during the past year by the fact that a German company was said to have discovered a process for making synthetic camphor, the report circulated being to the effect that the discovery while not yet perfect, gave promise of a cheaper product than that furnished by the monopoly. The Mikado's secret agents have been kept busy in order that the government might know just what progress was being made by this German company, and it is intimated that the almond-eyed monarch stood ready to pay a pretty sum to kill the European enterprise when it became a dangerous competitor.

And all this time, it now develops, an American company has been quietly making campohr, increasing its output monthly and improving the product until now it is turning out 500 pounds a day at a cost which enables it to sell at a price less than that quoted by the monopoly for the natural product. Whether the Mikado's secret agents will now transfer their attention to this country remains to be seen.—*Pacific Drug Review.*

**Inlays without a Matrix.** For the last eight years I have not used any matrix or used any metal when taking the impression and making the inlay. I proceed in this manner (I hope if you adopt it you will have the patience to learn it; it is very difficult):

For taking the impression I employ sticky wax, first softening it and then forcing it into the cavity, and from that impression I make my cast in powdered silex one-fifth part to five of plaster. But there is a good deal of difference in the powdered silex you get; I cannot use silex that is too fine. After making the model or counterpart of the impression, I cover it with liquid silex, very thin, and let it set twenty-four hours before baking. Before putting the porcelain into the mold I dry my cast thoroughly, warm it thoroughly, and then proceed in the ordinary manner of filling up a matrix and baking.

The result is that I have an inlay that comes out with silex at the back of it but faced by the poreclain, and I think it fills better; there is no space between the porcelain and the tooth. It was very difficult to arrive at success and I worked for two or three years before I was satisfied! but I have now been using this method for eight years very successfully.—*Cosmos.*

**Retaining Rubber Dam by Wire Ligatures.** From time to time in our literature it has been recommended to use the wire ligature instead of silk in connection with the application of the rubber dam, but this recommendation has not met with a wide adoption. The reason probably is that no suitable wire has ever been found. Recently an attempt was made to use such a ligature, making use of the ligature wire prepared for those who adopt the angle method for regulating teeth. The success was most gratifying and this angle ligature wire will be found extremely useful in many difficult cases where the cavity has a margin extending beneath the gum. The ligature wire can be applied and twisted firm and then pressed so as to expose all portions of the cavity, the wire remaining in place, which, of course, the silk would not do. Another mode of using the wire suggested by Dr. J. M. Thompson, of Detroit, Mich., also serves a useful purpose. In this mode the wire is first doubled into a loop and the loop end twisted so as to form a tight rope-like end. The two ends of the wire are then spread apart, bent around the tooth and twisted on the opposite side of the tooth to be filled. This is then cut off and we now have a twisted loop of wire on both the buccal and lingual aspects. These are so bent as to lie over against the tooth, the rubber is slipped on and the ends turned back. By this means the rubber can be held even on a short tooth where it would be difficult to use a clamp.—*Items of Interest.*

**Anesthesia in Face Operations.** Dr. Geo. W. Crile, Cleveland, Ohio, describes a method of administering the anesthetic and preventing the inhalation of blood in certain operations on the mouth and face, the steps of which are as follows:

1. The patient is reduced to full surgical anesthesia.
2. The pharynx is cocainized.
3. Two drainage-tubes as large as possible are passed through the nares to the level of the epiglottis; the tubes are then severed at an equal distance from the nose.
4. The mouth is well opened and the tongue drawn out.
5. The entire pharynx is then packed with rather large pieces of gauze.
6. If thoroughly done, the base of the tongue is carried well forward, and an air-chamber with which the rubber tubes and the larynx communicate is thereby formed.

The author considers that this method presents the following advantages:

1. The anesthetic is administered evenly and entirely away from the field of operation.
2. The patient may be placed in positions best suited to the operative technique, regardless of blood.
3. In operations upon the tongue there is sufficient pressure against the base usually to stop hemorrhage until the lingual arteries are cut.

In cleft palate operations the field of operation is cleaner and the mucus that usually forms in the throat is absorbed by the gauze.—*Annals of Surgery*.

**How Radium is Obtained.** In spite of the fact that the marvels of radium have been so widely discussed and have created such a flurry of excitement not only in the scientific world but among the general public, probably very few people are acquainted with the method by which it is secured in the minute quantities that are as yet available. That the element is obtained from pitch-blende is generally known, but some details of the exact process will be of interest. According to the *Lancet*, operations for the extraction are commenced by crushing the pitch-blende and then roasting the powder with carbonate of soda.

After washing the residue is treated with dilute sulphuric acid; then the sulphates are converted into carbonates by boiling with strong carbonate of soda. The residue contains radium sulphate, which is an exceedingly insoluble salt. The soluble sulphates are washed out, and the residue or insoluble portion is easily acted upon by hydrochloric acid, which takes out, among other things, polonium and actinium. Radium sulphate remains unattacked, associated with some barium sulphate. The sulphates are then coverted into carbonates by treatment with a boiling strong solution of carbonate of soda. The carbonates of barium and radium are next dissolved in hydrochloric acid and precipitated again as sulphates by means of sulphuric acid. The sulphates are further purified and ultimately converted into chlorides, until about fifteen pounds of barium and radium cholorid are obtained by acting upon one ton of crushed pitch-blende. Only a small fraction of this mixed chloride is pure radium chloride, which is finally separated from the barium chloride by crystallization, the crystals from the most radio-active of the solutions being selected. In this way the crystals ultimately obtained are relatively pure radium chloride of a very high degree of radio-activity.—*Stomatologist.*

**Age of Children Determined by Dentition.** Dr. B. F. Bell, of Aurora, Ills., presented the following suggestion for legislation to determine the ages of children who were put to work in factories before the proper age to the Northern Illinois Dental Society for discussion and suggestion:

"For an act to ascertain the age of children of the age of sixteen years or under and fourteen years and over.

"Section 1. Be it enacted by the people of the State of Illinois, represented in the General Assembly: It shall be lawful for any duly authorized dentist who is licensed to practice dental surgery in this State to issue a certificate of age for children of the age of sixteen years and under and of the age of fourteen years and over, which certificate shall set out the progress of dentition of such child, and shall state, in addition thereto, his opinon, from the best of his knowledge and belief, from general appearances, what the probable age of such child is.

"This certificate shall be made under affidavit and shall be accepted as sufficient proof of age in all cases where the age of such person is required in law."

The physician can not always swear that the child whose parents have put it to work is the one at whose birth he was present fourteen years before. Records of births in this country, and more especially in other countries, cannot always be verified, and Boards of Education may also be deceived as to a child's age. Hence the questions arise: Must or can dentition determine children's ages when other methods fail? Can it say with reasonable certainty when the child is of an age when he may be legally removed from school, or employed in factory or office? Will the fact that his dentition is more or less developed than his years warrant be proof that he is mentally and physically developed in proportion? And shall the child be put at labor or removed from school or become of age in the sight of the law hereafter, not according to his years, but according to his dentition?—February *Review*.

**English Dental Law and Consequences of Enforcement.**

Frederick Rose, L.D.S., in an address to the Liverpool Odontological Society, indicates by the following extract that the working of the English law is not more satisfactory than much of our legislation which was designed to control the practice of dentistry and to place it in the hands of truly professional men.

"We have evolved a certain code of ethical rules, the result of experience as to what will be for the greatest good of the greatest number, and have obtained the recognition by law of these rules. Now, these men I refer to want to play the game; but will not abide by the rules that govern it; and unfortunately, so far, we have not succeeded in doing much to get the legislature to help us to have them enforced.

"Nay more, the law has only, I fear, assisted in creating a still more formidable rivalry for us, and a menace to the well-being of the community, whose dental requirements it is for us to look after, than we should have had, had there been no Dentist's Act. Now, instead of having individuals to fight we have powerful companies, backed by much money and influence.

"There are some us, members of the British Dental Association, who are beginning to think that we are working on wrong lines in our endeavors to suppress this glaring outrage on our rights; that until we can get the law changed we are only sowing 'dragons' teeth.'"

There are two classes arrayed against the truly professional. First, there is the class consisting of men who having a certain measure of mechanical skill, and who, by advertising and low fees, and other unprofessional practices, attract the poor and ignorant. Secondly, the wealthy companies who are able to employ highly skilled but unscrupulous assistants, and who by buying their advertising in quantity get low rates and can therefore do much to attract from the professional and registered men patients they would otherwise get.

The former class can be easily suppressed; but when this is done they combine and form companies which the present law protects, and matters are no better, but worse.—*Record.*

**Reciprocity in Dental Registration.** Touching this question, just a few words: Reciprocity in dental registration is certain to be established, and in all probability at no very distant day; in fact, the forerunner of this is in evidence in the attention being given to the subject in our various publications. At first thought it would seem no more than fair and equable that if a man has been adjudged competent to practice in one State he ought to have the right to practice in any other State. As to interstate reciprocity, "this," as Dr. Brophy has recently said, undoubtedly is a good idea. If a man is able to meet the requirements of a State Board of Dental Examiners and is given a license to practice in that State by its board, it would appear that boards of other States should accept such credentials and grant their license without the formality of an examination. If the State has not a board which merits confidence of the board of other States, there is cause for the profession of that State to hold in question the subservience of its interests and reason for it to endeavor to bring about improvement. The real trouble is the lack of expressed uniformity in the requirements of the different States. And reciprocity of an indiscriminate character would undoubtedly have the effect of driving the applicants for license from a State demanding strict requirements to those holding lesser, which would certainly act to the detriment of dental education. It would prove a long step backward. However, this need not militate against reciprocity—between two or more States, of optional character—if the various requirements necessary for the granting of a license are practically uniform. In the medical profession reciprocity exists between the States of Wisconsin, Indiana, Michigan, Ohio, Iowa and Kansas. These States have formed what is known as "The American Federation of Reciprocating, Examining and Licensing Boards."

Universal reciprocity is desirable, but it will require considerable care in bringing it to a reality. In the meantime it would seem good judgment and be no more than right for the States holding these same requirements to grant reciprocal registration. And as soon as this Association can conscientiously feel this to be wise and beneficial it is hoped it will put itself upon record in favor of such a step.—R. S. Holden, in February *Review*.

